# Supplementary material for: The RNA-binding protein Puf5 and the HMGB protein Ixr1 regulate cell cycle-specific expression of CLB1 and CLB2 in Saccharomyces cerevisiae
Source: PLoS One. 2025 Feb 3;20(2):e0316433. doi: 10.1371/journal.pone.0316433 (PMC11790140; doi:10.1371/journal.pone.0316433)
Supplement: S1 Table — (DOCX) [file pone.0316433.s001.docx]

**S1 Table. The strains used in this study.**

| Strain | Genotype | Source |
| --- | --- | --- |
| 10BD | *MATa/MATα ade2/ade2 trp1/trp1 can1/can1 leu2/leu2 his3/his3 ura3/ura3* | 1 |
| 10BD-p5c2 | *MATa/MATα ade2/ade2 trp1/trp1 can1/can1 leu2/leu2 his3/his3 ura3/ura3 PUF5/puf5Δ::CgLEU2 CLB2/clb2Δ::CgHIS3* | 2 |
| 10BD-p5c1 | *MATa/MATα ade2/ade2 trp1/trp1 can1/can1 leu2/leu2 his3/his3 ura3/ura3 PUF5/puf5Δ::CgTRP1 CLB1/clb1Δ::CgHIS3* | 2 |
| 10BD-p5c1c2 | *MATa/MATα ade2/ade2 trp1/trp1 can1/can1 leu2/leu2 his3/his3 ura3/ura3 PUF5/puf5Δ::CgTRP1 CLB1/clb1Δ::CgLEU2 CLB2/clb2Δ::CgHIS3* | This study |
| 10BD-p5c1c2c6 | *MATa/MATα ade2/ade2 trp1/trp1 can1/can1 leu2/leu2 his3/his3 ura3/ura3 PUF5/puf5Δ::CgTRP1 CLB1/clb1Δ::CgLEU2 CLB2/clb2Δ::CgHIS3 CLB6/clb6Δ::CgLEU2* | This study |
| 10BD-p5c5c6 | *MATa/MATα ade2/ade2 trp1/trp1 can1/can1 leu2/leu2 his3/his3 ura3/ura3 PUF5/puf5Δ::CgTRP1 CLB5/clb5Δ::CgHIS3 CLB6/clb6Δ::CgLEU2* | This study |
| 10BD-d1c2i1 | *MATa/MATα ade2/ade2 trp1/trp1 can1/can1 leu2/leu2 his3/his3 ura3/ura3 DUN1/dun1Δ::CgTRP1 CLB2/clb2Δ::CgHIS3 IXR1/ixr1Δ::CgLEU2* | This study |
| 10BD-d1c2i1s1 | *MATa/MATα ade2/ade2 trp1/trp1 can1/can1 leu2/leu2 his3/his3 ura3/ura3 DUN1/dun1Δ::CgTRP1 CLB2/clb2Δ::CgHIS3 SML1/sml1Δ::CgLEU2 IXR1/ixr1Δ::KlURA3* | This study |
| 10BD-m1c2sl | *MATa/MATα ade2/ade2 trp1/trp1 can1/can1 leu2/leu2 his3/his3 ura3/ura3 MEC1/mec1Δ::CgTRP1 CLB2/clb2Δ::CgHIS3 SML1/sml1Δ::CgLEU2* | This study |
| 10BD-d1c5c6 | *MATa/MATα ade2/ade2 trp1/trp1 can1/can1 leu2/leu2 his3/his3 ura3/ura3 DUN1/dun1Δ::CgTRP1 CLB5/clb5Δ::CgHIS3 CLB6/clb6Δ::CgLEU2* | This study |
| 10BD-p5d1c5c6 | *MATa/MATα ade2/ade2 trp1/trp1 can1/can1 leu2/leu2 his3/his3 ura3/ura3 PUF5/puf5Δ::CgTRP1 DUN1/dun1Δ::CgHIS3 CLB5/clb5Δ::KlURA3 CLB6/clb6Δ::CgLEU2* | This study |
| 10BD-d1c1i | *MATa/MATα ade2/ade2 trp1/trp1 can1/can1 leu2/leu2 his3/his3 ura3/ura3 DUN1/dun1Δ::CgTRP1 CLB1/clb1Δ::CgTRP1 IXR1/ixr1Δ::CgLEU2* | This study |
| WT | *MATa ade2 trp1 can1 leu2 his3 ura3* | 2 |
| WT | *MATα ade2 trp1 can1 leu2 his3 ura3* | 2 |
| *puf5∆* | *MATα ade2 trp1 can1 leu2 his3 ura3 puf5Δ::CgTRP1* | 2 |
| *puf5∆* | *MATa ade2 trp1 can1 leu2 his3 ura3 puf5Δ::CgTRP1* | 2 |
| *puf5∆* | *MATa ade2 trp1 can1 leu2 his3 ura3 puf5Δ::CgHIS3* | 2 |
| *clb2∆* | *MATα ade2 trp1 can1 leu2 his3 ura3 clb2Δ::CgLEU2* | 2 |
| *ixr1∆* | *MATα ade2 trp1 can1 leu2 his3 ura3 ixr1Δ::CgLEU2* | 2 |
| *bar1∆* | *MATa ade2 trp1 can1 leu2 his3 ura3 bar1∆::CgHIS3* | 2 |
| *bar1∆*  *puf5∆* | *MATa ade2 trp1 can1 leu2 his3 ura3 bar1∆::CgHIS3 puf5Δ::CgTRP1* | 2 |
| *bar1∆*  *ixr1∆* | *MATa ade2 trp1 can1 leu2 his3 ura3 bar1∆::CgHIS3 puf5Δ::CgTRP1 ixr1Δ::CgLEU2* | 2 |
| *bar1∆*  *puf5∆*  *ixr1∆* | *MATa ade2 trp1 can1 leu2 his3 ura3 bar1∆::CgHIS3 puf5Δ::CgTRP1 ixr1Δ::CgLEU2* | 2 |
| *puf5∆ clb2∆* | *MATα ade2 trp1 can1 leu2 his3 ura3 puf5Δ::CgTRP1 clb2Δ::CgHIS3* | 2 |
| *puf5∆ ixr1∆* | *MATα ade2 trp1 can1 leu2 his3 ura3 puf5Δ::CgTRP1 ixr1Δ::CgLEU2* | 2 |
| *puf5∆ clb2∆ ixr1∆* | *MATα ade2 trp1 can1 leu2 his3 ura3 puf5Δ::CgTRP1 clb2Δ::CgHIS3 ixr1Δ::CgLEU2* | 2 |
| *clb1∆ clb5∆ clb6∆* | *MATα ade2 trp1 can1 leu2 his3 ura3 clb1Δ-clb6∆::CgLEU2 clb5Δ::CgHIS3* | This study |

**References**

1. Tadauchi T, Matsumoto K, Herskowitz I, Irie K. Post-transcriptional regulation through the *HO* 3’-UTR by Mpt5, a yeast homolog of Pumilio and FBF. EMBO J. 2001; 20: 552–561. doi.org/10.1093/emboj/20.3.552

2. Sato M, Irie K, Suda Y, Mizuno T, Irie K. The RNA-binding protein Puf5 and the HMGB protein Ixr1 contribute to cell cycle progression through the regulation of cell cycle-specific expression of CLB1 in Saccharomyces cerevisiae. PLoS Genet. 2022;18: e1010340. doi: 10.1371/journal.pgen.1010340. PMID: 35905103; PMCID: PMC9365169.
