# Supplementary material for: The RNA-binding protein Puf5 and the HMGB protein Ixr1 regulate cell cycle-specific expression of CLB1 and CLB2 in Saccharomyces cerevisiae
Source: PLoS One. 2025 Feb 3;20(2):e0316433. doi: 10.1371/journal.pone.0316433 (PMC11790140; doi:10.1371/journal.pone.0316433)
Supplement: S2 Table — (DOCX) [file pone.0316433.s002.docx]

**S2 Table. Plasmids used in this study**

| Plasmids | Relevant markers | Reference |
| --- | --- | --- |
| YCplac33 | *URA3, CEN-ARS* | 1 |
| YEplac195 | *URA3* | 1 |
| YEplac195-PUF5 | *URA3, PUF5* | This study |
| YEplac195-CLB1 | *URA3, CLB1* | 2 |
| YEplac195-CLB2 | *URA3, CLB2* | 2 |
| YEplac195-CLB5 | *URA3, CLB5* | This study |
| YEplac195-CLB6 | *URA3, CLB6* | This study |
| YCplac33-CLB2 | *URA3, CEN-ARS, CLB2* | This study |
| YCplac33-IXR1 | *URA3, CEN-ARS, IXR1* | 2 |
| YCplac33-CLB1-3HA-CLB1 3’UTR | *URA3, CEN-ARS, CLB1-3HA* | 3 |
| YCplac33-CLB2-3HA-CLB2 3’UTR | *URA3, CEN-ARS, CLB2-3HA* | 3 |
| pCgLEU2 | *C. glabrata LEU2* in pUC19 | 4 |
| pCgHIS3 | *C. glabrata HIS3* in pUC19 | 4 |
| pCgTRP1 | *C. glabrata TRP1* in pUC19 | 4 |
| pKlURA3 | *K. lactis URA3* in pUC19 | 4 |

**References**

1. Gietz RD, Sugino A. New yeast-Escherichia coli shuttle vectors constructed with in vitro mutagenized yeast genes lacking six-base pair restriction sites. *Gene*. 1988;74(2):527-534. doi:10.1016/0378-1119(88)90185-0

2. Sato M, Irie K, Suda Y, Mizuno T, Irie K. The RNA-binding protein Puf5 and the HMGB protein Ixr1 contribute to cell cycle progression through the regulation of cell cycle-specific expression of CLB1 in Saccharomyces cerevisiae. PLoS Genet. 2022;18: e1010340. doi: 10.1371/journal.pgen.1010340. PMID: 35905103; PMCID: PMC9365169.

3. Revilleza JEC, Sato M, Irie K, Suda Y, Mizuno T, Irie K. Regulation of CLB6 expression by the cytoplasmic deadenylase Ccr4 through its coding and 3' UTR regions. PLoS One. 2022 May 6;17(5):e0268283. doi: 10.1371/journal.pone.0268283. PMID: 35522675; PMCID: PMC9075657.

4. Sakumoto N, Mukai Y, Uchida K, Kouchi T, Kuwajima J, Nakagawa Y, et al. A series of protein phosphatase gene disruptants in *Saccharomyces cerevisiae*. Yeast. 1999; 15: 1669-1679. doi:10.1002/(SICI)1097-0061(199911)15:15<1669::AID-YEA480>3.0.CO;2-6
