# Supplementary material for: The RNA-binding protein Puf5 and the HMGB protein Ixr1 regulate cell cycle-specific expression of CLB1 and CLB2 in Saccharomyces cerevisiae
Source: PLoS One. 2025 Feb 3;20(2):e0316433. doi: 10.1371/journal.pone.0316433 (PMC11790140; doi:10.1371/journal.pone.0316433)
Supplement: S3 Table — (DOCX) [file pone.0316433.s003.docx]

**S3 Table. Primers used for the gene deletion.**

| Gene | Forward primer | Reverse primer |
| --- | --- | --- |
| *PUF5* | TTCTACGCAAATTTATAAATCAATTACGATTTTTCCAGTTTCTCTTCACAGGAAACAGCTATGACC | AATATTTGTACAGTAAGAAGGAAAGAAAAAGAAAGAAAAAAAAGTAGTTGTAAAACGACGGCCAGT |
| *CLB2* | AAGCCTTTTATTGATTACCCCCTCTCTCTCTTCATTGATCTTATAGCACAGGAAACAGCTATGACC | ATTTATCGATTATCGTTTTAGATATTTTAAGCATCTGCCCCTCTTCGTTGTAAAACGACGGCCAGT |
| *CLB1* | TTCGTCCGTTATATCAACCATCAAAGGAAGCTTTAATCTTCTCATACACAGGAAACAGCTATGACC | TAAAGTAAGGAAGTGAGATTTTGGTTTTCTGTGTAGGCTAGCACCTGTTGTAAAACGACGGCCAGT |
| *IXR1* | TCCATTCTGTGATATACGTACGACGCTAACAGTACCCACAACTGCACACAGGAAACAGCTATGACC | TTTGCGTGGGATAATGTTACAGTGGAAAACTAAAGTTGTTTATTTGGTTGTAAAACGACGGCCAGT |
| *BAR1* | CCTAAAATCATACCAAAATAAAAAGAGTGTCTAGAAGGGTCATATACACAGGAAACAGCTATGACC | TATATTTGATATTTATATGCTATAAAGAAATTGTACTCCAGATTTCGTTGTAAAACGACGGCCAGT |
| *CLB5* | CGCGCTTTTCCCTGTATTTAAAGCCGCTGAACACCTTTACTGAACACACAGGAAACAGCTATGACC | GAAAATGTAAAGAGTATGCGAATTCATGAGCATTACTAGTACTAATGTTGTAAAACGACGGCCAGT |
| *CLB6* | AAATTATTATTCTCTGATATTCTCTCCCTCCTTTTAAATTTTTAAACACAGGAAACAGCTATGACC | TATTTAAGATGCAGGGGGTTAGCTGGCTATAATTTTGATCTATGTTGTTGTAAAACGACGGCCAGT |
| *DUN1* | AAGTAAAGGGGCTTAACATACAGTAAAAAAGGCAATTATAGTGAAGCACAGGAAACAGCTATGACC | AAAAATCCAGATTCAAACAATGTTTTTGAAATAATGCTTCTCATGTGTTGTAAAACGACGGCCAGT |
| *SML1* | TTACGGTCTCACTAACCTCTCTTCAACTGCTCAATAATTTCCCGCTCACAGGAAACAGCTATGACC | ACTAGTGGGAAATGGAAAGAGAAAAGAAAAGAGTATGAAAGGAACTGTTGTAAAACGACGGCCAGT |
| *MEC1* | TGGACAACAAGAACGACATACACCGCGTAAAGGCCCACAAGACTGCCACAGGAAACAGCTATGAC | TAGATCAAGAGGAAGTTCGTCTGTTGCCGAAAATGGTGGAAAGTCGGTTGTAAAACGACGGCCAGT |
