# Supplementary material for: The RNA-binding protein Puf5 and the HMGB protein Ixr1 regulate cell cycle-specific expression of CLB1 and CLB2 in Saccharomyces cerevisiae
Source: PLoS One. 2025 Feb 3;20(2):e0316433. doi: 10.1371/journal.pone.0316433 (PMC11790140; doi:10.1371/journal.pone.0316433)
Supplement: S4 Table — (DOCX) [file pone.0316433.s004.docx]

**S4 Table. Primers used for the qRT-PCR.**

|  | Forward primer | Reverse primer |
| --- | --- | --- |
| *SCR1* | AACCGTCTTTCCTCCGTCGTAA | CTACCTTGCCGCACCAGACA |
| *ACT1* | TGCCGAAAGAATGCAAAAGG | TCTGGAGGAGCAATGATCTTGA |
| *CLB1* | TTCCGAGCAAGAAAAGCAGC | TCGTACTCCTCCAGAACCTC |
| *CLB2* | GCCGATGACTTCACCTCCTC | CTGCTGCTTTTCTTGCTCGG |
| *CLB3* | GGAACAAGAGGAACCCGTTG | AATTCGGCAACCATGACCAC |
| *CLB4* | CAGCAGATTCAAGCCGATGA | AGCGTACTCCACAACCATCA |
| *CLB5* | ACGGCAGCAGAGCAAGAAGA | TCTAGGTCCTGCCAGCCTAC |
| *CLB6* | GCGATCAACCTGCTAGATCG | ACGAATAGCTCAGCCTTCCT |
| *RNR1* | CCATGGCACCAATGCCTACT | CACCGGATAAGACACGACGG |
| *SIC1* | GGCTTACGTCTCCTCAACGC | CGACCCAATGGTTCCTGCTC |
| *RNR2* | ATGCTTGTTGTTCGCCCATTTG | TCAATTTCCACAGCCTCGGTG |
| *RNR3* | CAGCATTTGGTAATGCGTGTGGC | GTTGGGGAAGCGTGAGTGAAGTATC |
| *RNR4* | TCGAATTCATGGAGGATGTCGC | TGTCACTGGCCTTTTGGTAGTC |
