# Supplementary figures and images for: The RNA-binding protein Puf5 and the HMGB protein Ixr1 regulate cell cycle-specific expression of CLB1 and CLB2 in Saccharomyces cerevisiae
Source: PLoS One. 2025 Feb 3;20(2):e0316433. doi: 10.1371/journal.pone.0316433 (PMC11790140; doi:10.1371/journal.pone.0316433)

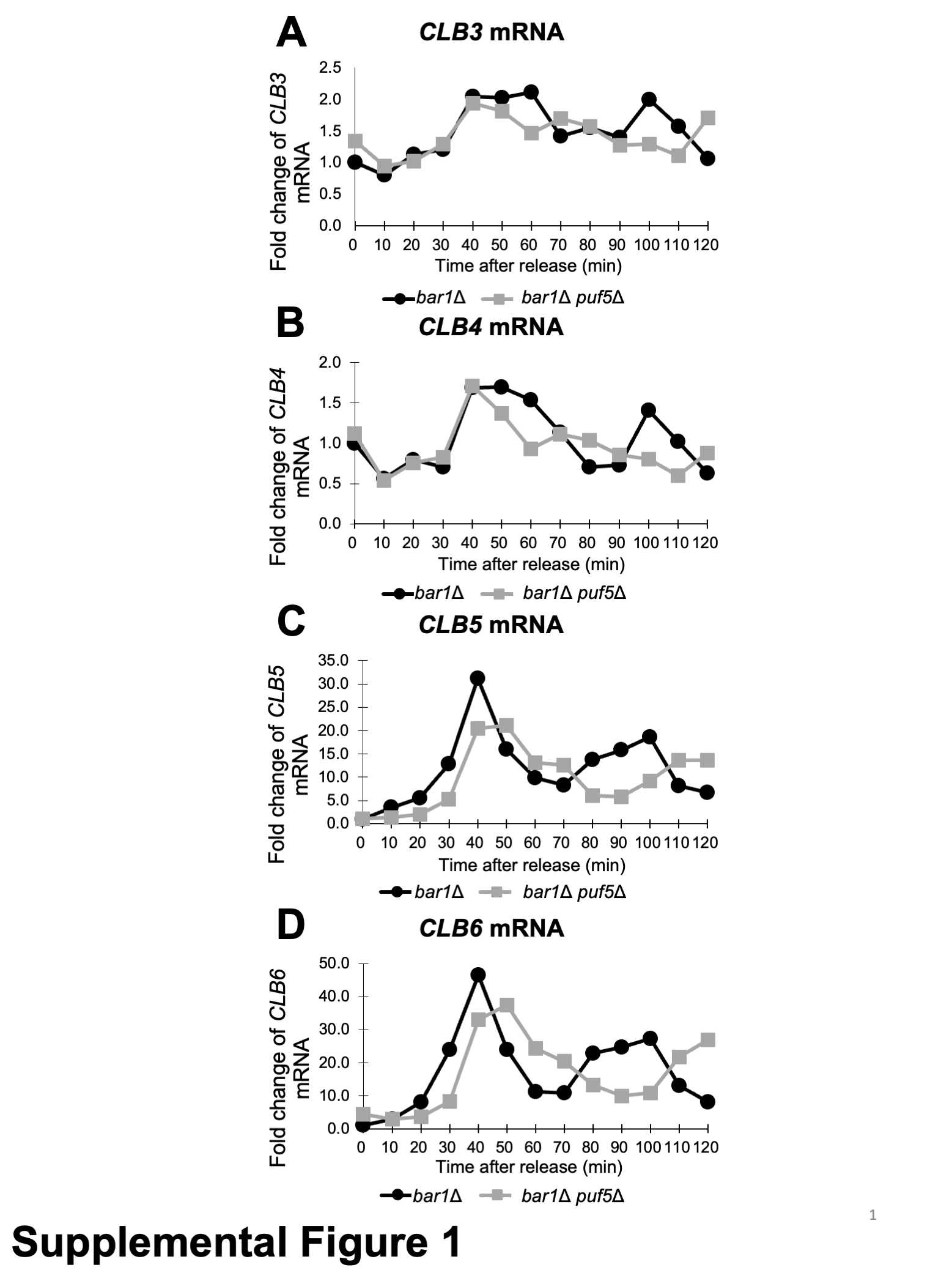

Supplement: S1 Fig — (A-D) The cell cycle-dependent mRNA levels of CLB3, CLB4, CLB5, and CLB6 in the synchronized bar1Δ cell (black circle) and bar1Δ puf5Δ mutant (grey square). The levels of CLB3 mRNA (A), CLB4 mRNA (B), CLB5 mRNA (C), and CLB6 mRNA (D) were quantified by qRT-PCR analysis, and the relative mRNA levels were calculated using the SCR1 reference gene. The vertical axis shows the fold change of mRNA level relative to that in the bar1Δ 0 min sample, and the horizontal axis shows the time after release from the G1-phase. (TIFF) [file pone.0316433.s005.tiff]

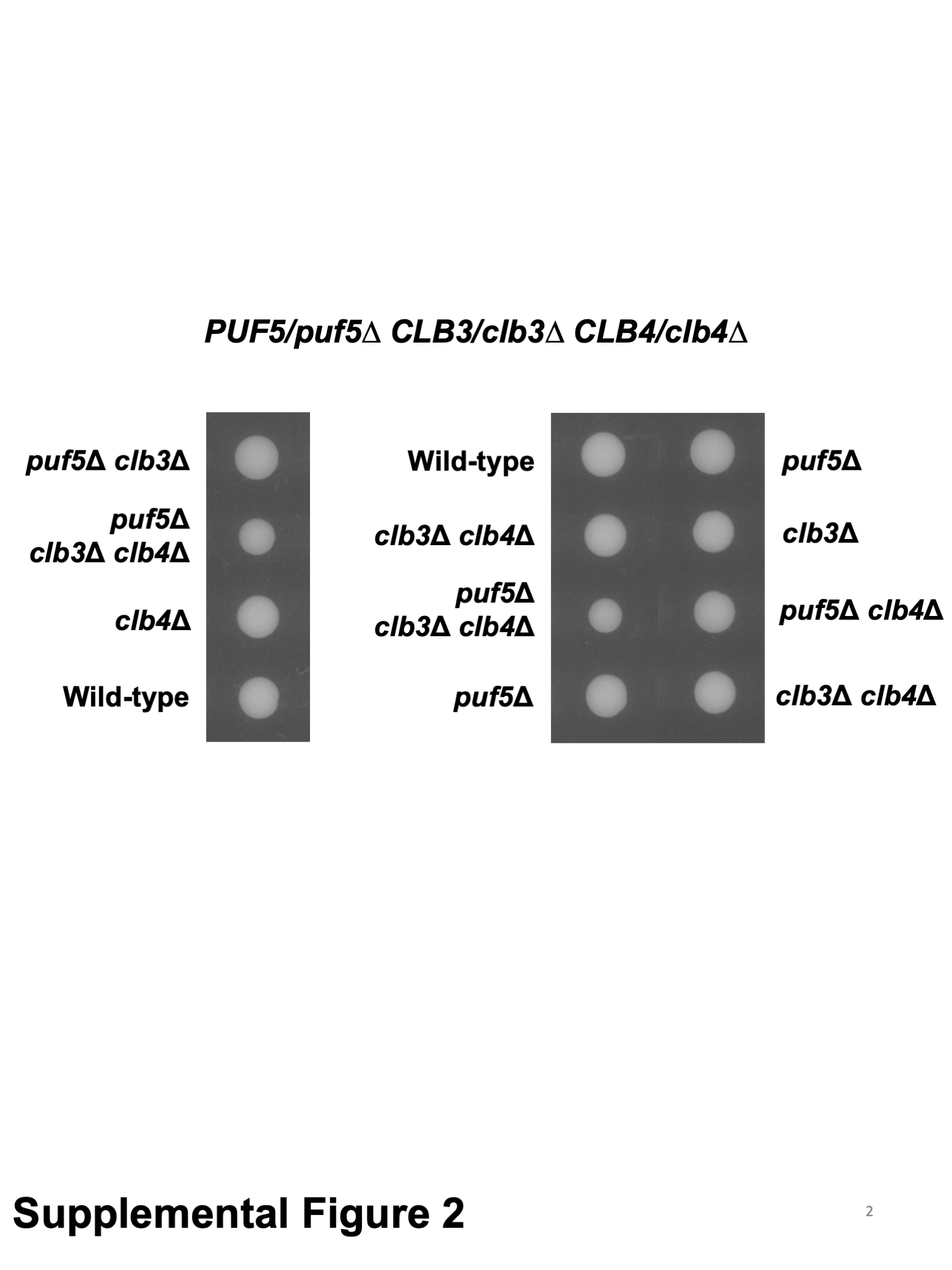

Supplement: S2 Fig — The tetrad analysis of the strains that are heterozygous for the alleles of PUF5, CLB3, and CLB4. The cells were sporulated, dissected on a YPD plate, and cultured at 30°C for 3 days. (TIFF) [file pone.0316433.s006.tiff]

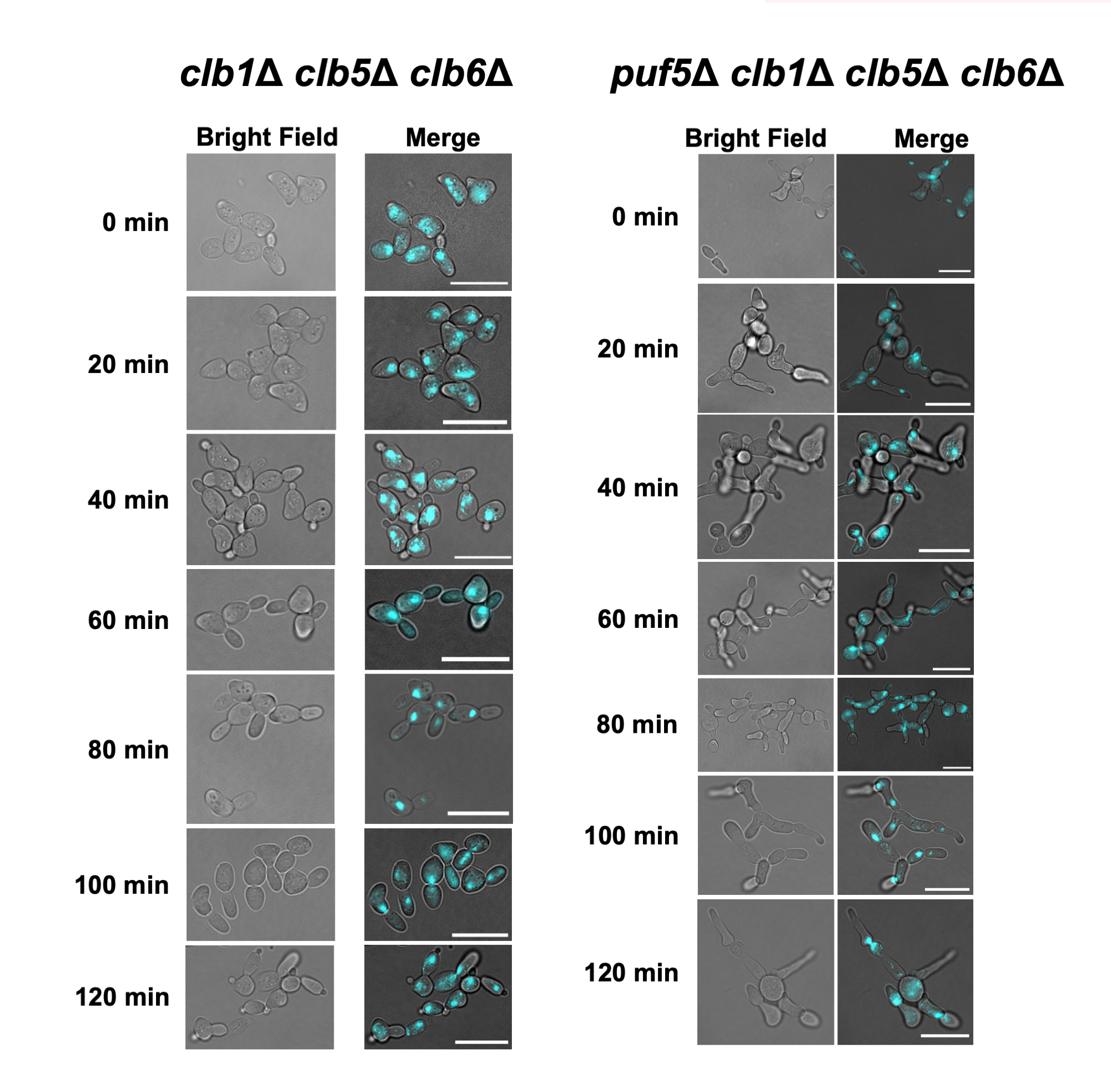

Supplement: S3 Fig — Morphology and nuclear images of the clb1Δ clb5Δ clb6Δ triple mutant (A) and the puf5Δ clb1Δ clb5Δ clb6Δ quadruple mutant (B). Cells were synchronously cultured and collected as described in the material and method section. Bright-field (left) and the overlayed (right) were shown. The scale bar represents 2 μm. (TIFF) [file pone.0316433.s007.tiff]

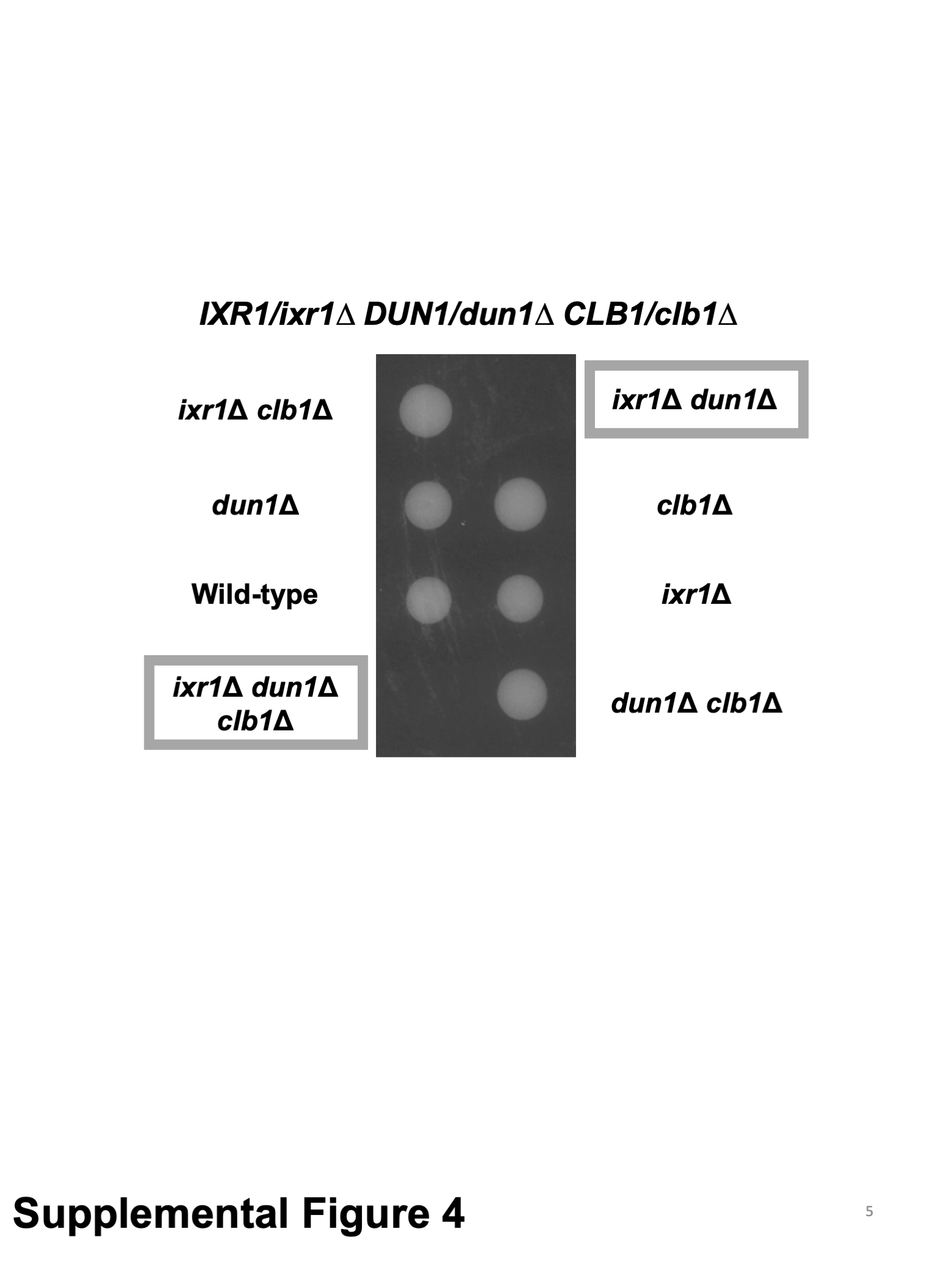

Supplement: S4 Fig — The tetrad analysis of the strains that are heterozygous for the alleles of IXR1, DUN1, and CLB1. The cells were sporulated, dissected on a YPD plate, and cultured at 30°C for 3 days. (TIFF) [file pone.0316433.s008.tiff]

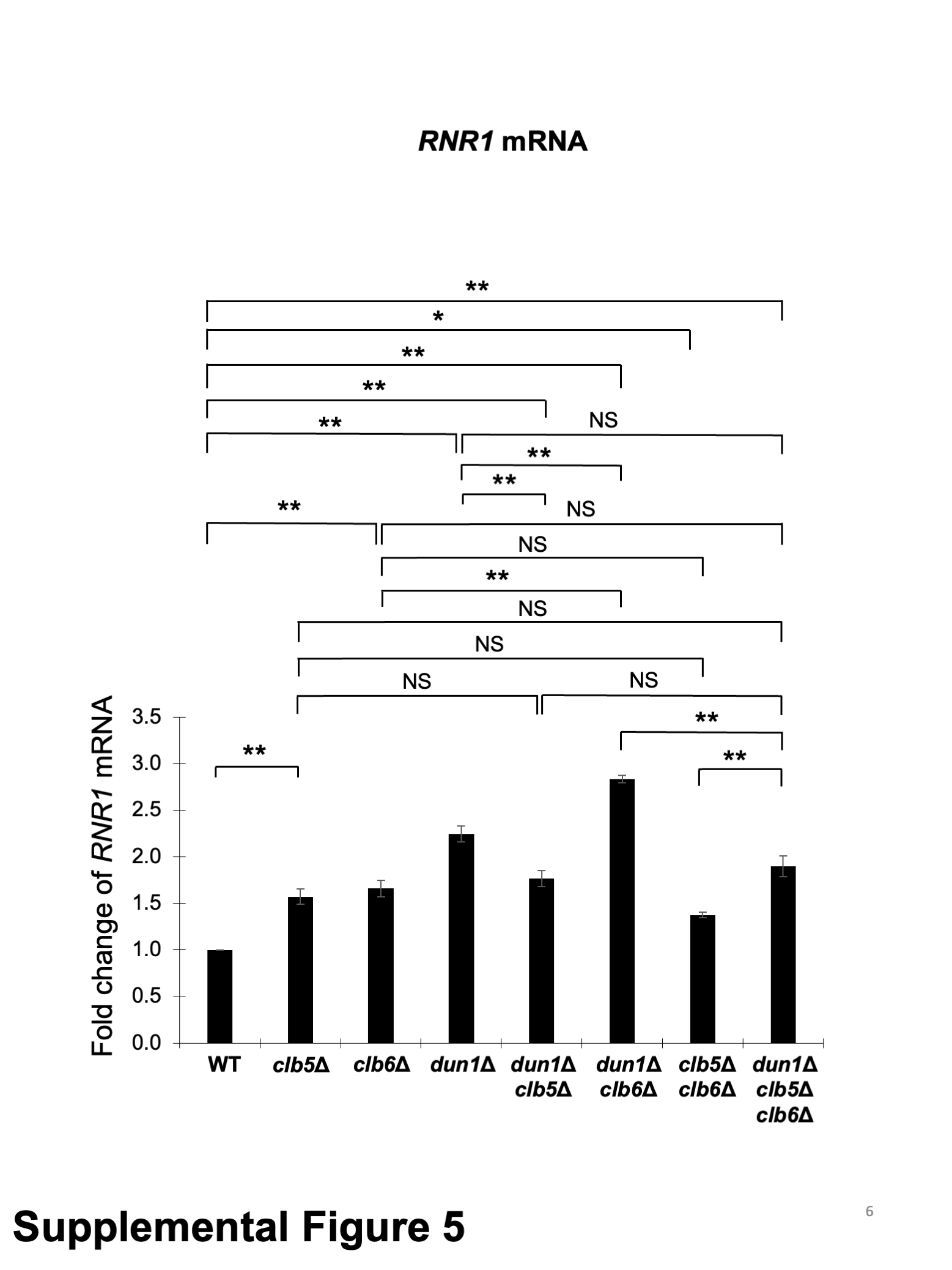

Supplement: S5 Fig — The mRNA levels of RNR1 in the wild-type strain, the dun1Δ mutant, the clb5Δ mutant, the clb6Δ mutant, the dun1Δ mutant, the dun1Δ clb5Δ mutant, the dun1Δ clb6Δ mutant, the clb5Δ clb6Δ mutant, and the dun1Δ clb5Δ clb6Δ mutant. The cells were cultured in a YPD medium at 28°C until the log phase. The RNR1 mRNA levels were quantified by qRT-PCR analysis, and the relative mRNA levels were calculated using the ACT1 reference gene. The data shows the mean ± SE (n = 3) of the fold change of RNR1 in relative to the mRNA level in the wild-type strain. *P < 0.05, **P < 0.01 as determined by Tukey’s test. NS indicates no significant change. (TIFF) [file pone.0316433.s009.tiff]

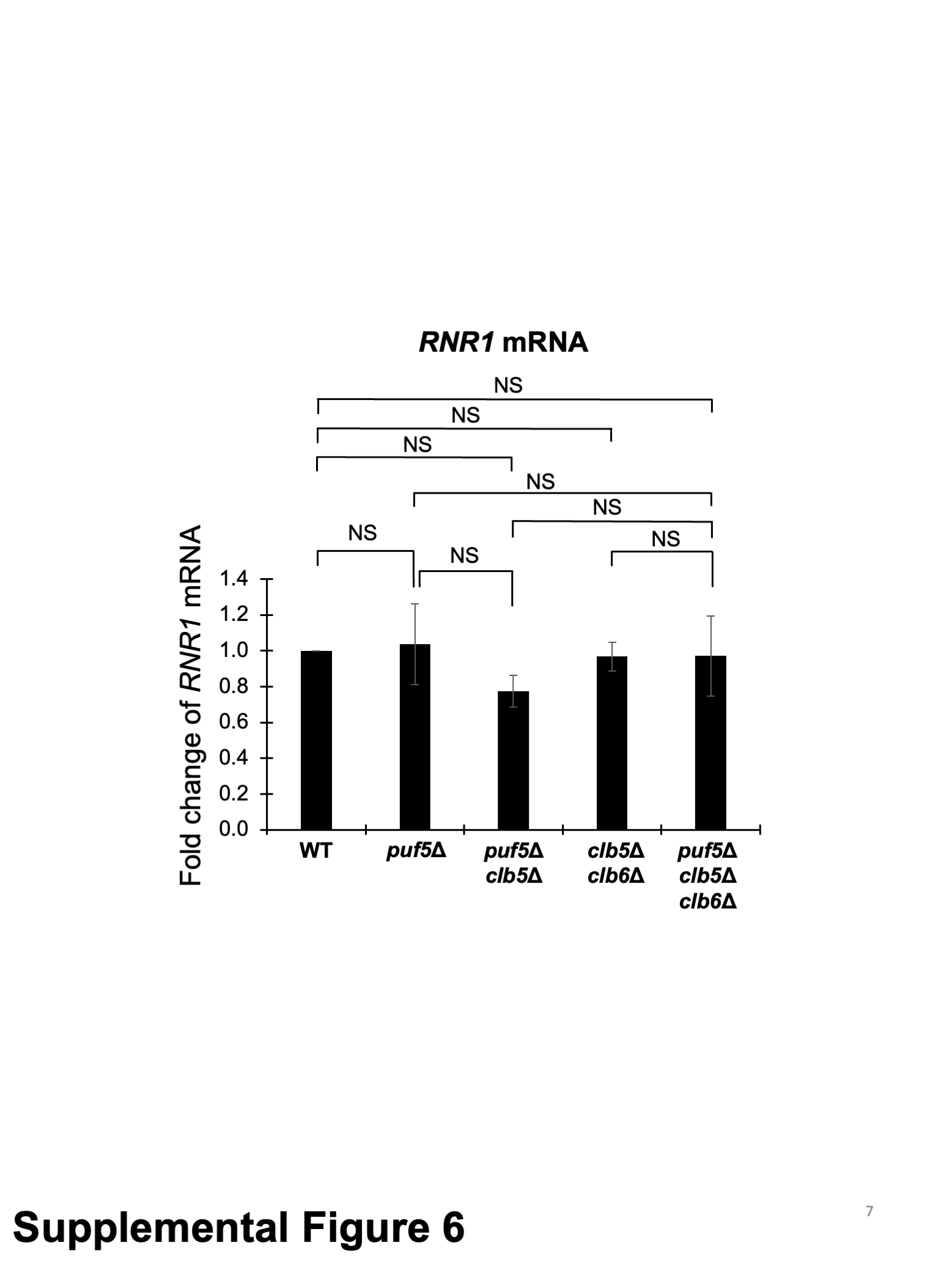

Supplement: S6 Fig — The mRNA levels of RNR1 in the wild-type strain, the puf5Δ mutant, the puf5Δ clb5Δ mutant, the clb5Δ clb6Δ mutant, and the puf5Δ clb5Δ clb6Δ mutant. The cells were cultured in a YPD medium at 28°C until the log phase. The RNR1 mRNA levels were quantified by qRT-PCR analysis, and the relative mRNA levels were calculated using the ACT1 reference gene. The data shows the mean ± SE (n = 3) of the fold change of RNR1 relative to the mRNA level in the wild-type strain. NS indicates no significant change. (TIFF) [file pone.0316433.s010.tiff]
