# Supplementary material for: The RNA-binding protein Puf5 and the HMGB protein Ixr1 regulate cell cycle-specific expression of CLB1 and CLB2 in Saccharomyces cerevisiae
Source: PLoS One. 2025 Feb 3;20(2):e0316433. doi: 10.1371/journal.pone.0316433 (PMC11790140; doi:10.1371/journal.pone.0316433)
Supplement: Attachment — Submitted filename: SatoRana_response-to-reviewers_20241126.docx [file pone.0316433.s012.docx]

**Our responses to the reviewers’ comments and changes in the revised manuscript**

Title : The RNA-binding protein Puf5 and the HMGB protein Ixr1 regulate cell cycle-specific expression of *CLB1* and *CLB2* in *Saccharomyces cerevisiae*

Authors: Megumi Sato, Varsha Rana, Yasuyuki Suda, Tomoaki Mizuno, Kenji Irie

**Reviewer #1**

**1. Please provide the number of experiments conducted or the biological replicates for Figures 3, 4, 5, and 6 in the figure legends.**

For all cell cycle experiments, we performed the experiments two times and presented the representative data. As for the experiment for Figure 2 in the revised manuscript (Figure 3 in the first edition), we isolated RNA from the same culture for Figure 3A and observed similar expression patterns to Figures 2A-2D by qRT-PCR analysis. In addition, for each experiment for Figures 5, 6, and 8 in the revised manuscript (Figures 4, 5, and 6 in the first edition, respectively), we repeated the same experiment and obtained similar results. Therefore, we presented the representative data for each figure and noted in the Figure legend.

**2. The authors should carefully review the entire text.**
According to the reviewer’s comment, we thoroughly reviewed and re-wrote the entire manuscript.

**Lines 177 and 178: “the expression…CLB5 and CLB6 remained unchanged”. According to Supplemental Figure 1, the expressions of CLB5 and CLB6 were slightly down-regulated, and their expression timing was shifted to later.**

The expression of *CLB5* and *CLB6* was actually decreased slightly in the *bar1∆ puf5∆* double mutant than that in the *bar1∆* mutant as reviewer pointed. However, the extent was much lower than the downregulation of *CLB1* and *CLB2* expressions. Therefore, we considered the effect on *CLB1*/*CLB2* significant, but not on *CLB5*/*CLB6.* We mentioned the results in lanes 175-179, page 9, of the revised manuscript.

**Lines 243 and 244: “the puf5Δ clb5Δ double…by the multi-copy CLB2, CLB5, and CLB6”. In Figure 9A, complementation with YEpCLB6 at 37°C was not sufficient.**

In Figure 10A in the revised figures, the *puf5∆ clb5∆* double mutant strain harboring YEp*CLB6* grew slightly better than the strain with a vector at 37℃, but this suppression was far weaker than *CLB5* or *CLB2.* Therefore, from these results, we assume that the effect of the multi-copy *CLB6* is far milder than that of the multi-copy *CLB2* or *CLB5.* We included the detailed explanation of these results in lanes 290-294, page 14, in the revised manuscript.

**Lines 269 and 270: “a single copy or…inhibited the growth of the ixr1∆ dun1∆ clb2∆”. The suppression of growth by a single copy CLB2 plasmid was very mild (Figure 10B).**

As for the inhibitory effect of a single-copy *CLB2*, we carefully interpret the results and concluded that the effect was milder than that of a multi-copy *CLB2*, and that controlling the dosage of Clb2 was essentially important for the cell survival in the *dun1∆* background. We described the results in lanes 330-335, page 15, and discussed the dosage effect of Clb2 in lanes 463-470, page 21, in the discussion section of the revised manuscript.

**Lines 298 and 299: “the dun1Δ clb6Δ double…slow growth”. It is difficult to see the slow-growth phenotype of that mutant (Figure 13A).**

We cautiously interpreted the data and reasoned out that the *dun1∆ clb6∆* double mutant did not show growth retardation. Reflecting that, we revised the manuscript and described the data in lanes 376-378, page 17.

**Lines 317-319: The last sentence of the results section is purely speculative, so it might be better to move it to the discussion section.**

In response to the reviewer’s suggestion, we moved the sentences mentioning to the relationship between *RNR3* expressions and DNA damage into discussion section. We discussed the hypothesis about DNA damage in lanes 483-509, pages 22-23.

**Lines 374-381: The sentences may need to be reviewed for clarity regarding the author’s claim.**

According to the revision of the manuscript, we re-wrote whole sentences in the discussion section carefully considering our data.

**Reviewer #2**

**The study proposes a mechanism for Puf5's regulation of the cell cycle by demonstrating the degree of yeast growth and qRT-PCR results using genetics. However, it remains unclear whether the expression levels of cyclin mRNA and cyclin proteins are correlated. To strengthen the validity of the study, it would be helpful to show protein expression levels of Clb1 and Clb2, particularly in Figure 1 and Figures 3C, D at the 60-80 min time points, which are critical to the study.**

According to the reviewer’s suggestion, we examined the 3HA-tagged Clb1 and Clb2 protein levels in the *bar1∆* mutant or the *bar1∆ puf5∆* double mutant throughout cell cycle. For this experiment, we transformed a single-copy *CLB1*-3HA or a *CLB2*-3HA plasmid into the two strains and collected samples as described in the material and method section. Consequently, as shown in Figure 3, we observed a significant decrease in the Clb1 protein levels in the *bar1∆ puf5∆* double mutant than that in the *bar1∆* mutant, even though the basal Cl1 protein levels were low. Therefore, the downregulation of *CLB1* mRNA levels seem to be reflected in the protein levels. As for the Clb2, the protein level was mildly decreased at 40 min time point in the *bar1∆ puf5∆* double mutant compared to the *bar1∆* mutant. However, the Clb2 protein levels did not show significant difference between the two mutants after 60 min time point. Nevertheless, regarding the data at 40 min time point, we assume that we could confirm the delay in the induction of Clb2 protein expression in the *puf5∆* strains. We described the results in lanes 154-170, page 8, in the revised manuscript.

For one reason why Clb2 protein levels were not significantly decreased in the *puf5∆* strains as mRNA level, we hypothesize that there are some compensatory mechanisms for the Clb2 protein levels, since Clb2 is the most important mitotic cyclin. We described this in lanes 164-167, page 8, in the revised manuscript.

Nevertheless, it is certain that the downregulation of *CLB2* expression in the *puf5∆* strains, albeit very mild, physiologically important in the deficiency of S-phase cyclins. Therefore, we regard the observed delay in the Clb2 induction to be significant and important for cell growth.

**Lines 218-221: The delay in SIC1 mRNA expression in the puf5Δ clb1Δ clb5Δ clb6Δ quadruple mutant is attributed to a G2 phase delay, but it is also possible that cell cycle synchronization is inconsistent. It would be advisable to soften the expression in the manuscript or support the authors' conclusions by performing nuclear and cell wall staining followed by microscopic observations.**

We observed the morphology and nuclei of the *clb1∆ clb5∆ clb6∆* triple mutant and the *puf5∆ clb1∆ clb5∆ clb6∆* quadruple mutant after releasing from G1-phase arrest using nuclear staining by DAPI. In both mutants, cell cycle progression was successfully observed from morphological changes and nuclei behaviors, even though the *puf5∆ clb1∆ clb5∆ clb6∆* quadruple mutant showed highly elongated phenotype. We also observed that the *puf5∆ clb1∆ clb5∆ clb6∆* quadruple mutant was not able to complete M-phase. Therefore, consistent with the data in Figure 8C that *SIC1* expression was not induced in the quadruple mutant, the mutant was suggested to have difficulties in accomplishing the mitosis. We presented microscopic data in Figure S3A and S3B and mentioned in lanes 251-266, pages 12-13, in the revised manuscript.

**Lines 225-227: Both CLB2 overexpression and PUF5 overexpression suppress the growth defect of the puf5Δ clb1Δ clb5Δ clb6Δ strain, but PUF5 overexpression appears to promote faster growth. Could this be due to PUF5 overexpression regulating target molecules other than CLB2? Alternatively, when using a plasmid, the cell cycle-dependent expression of Clb2 may not be fully replicated, and plasmid-derived Clb2 might not function as effectively as endogenous Clb2. Since PUF5 overexpression regulates endogenous Clb2 expression, it could restore the cell cycle-dependent expression pattern of Clb2, leading to better recovery of Clb2 function than CLB2 overexpression. Current data do not clarify whether CLB2 expressed from a plasmid exhibits cell cycle-dependent expression, making it difficult to determine whether CLB2 overexpression is fully functional. Including clb1Δ clb5Δ clb6Δ-YEplac195 as a control would allow for an evaluation of whether the effects of exogenously expressed CLB2 overexpression are complete or partial.**

In the experiment for Figure 3B, strains harboring a YCplac33-*CLB2*-3HA plasmid showed rhythmical expression patterns of Clb2-3HA protein. Therefore, we assume that the plasmid-induced expression of *CLB2* is also regulated in a cell cycle specific manner. Actually, multi-copy *PUF5* suppressed the growth defect of the *puf5∆ clb1∆ clb5∆ clb6∆* quadruple mutant stronger than multi-copy-*CLB2*, but we do not assume that these results originated from the difference of expressional patterns between plasmid-induced *CLB2* and endogenous *CLB2*. Rather, considering that Puf5 is a multi-functional RNA binding protein, we hypothesize that the regulation of other target genes by Puf5 contributed to the phenotype. We discussed this suggestion in lanes 428-433, page 20, in the revised manuscript.

**Lines 265-266: Does CLB1 play a role in the growth defect of ixr1Δ dun1Δ? Would the ixr1Δ dun1Δ clb1Δ clb2Δ strain show improved growth compared to the triple mutant, ixr1Δ dun1Δ clb2Δ? To discuss the functional distinction between Clb1 and Clb2, it would be preferable to show the effects of clb2Δ on ixr1Δ dun1Δ.**

For clarifying the contribution of *CLB1* expression to the growth defect of the *dun1∆ ixr1∆* double mutant, we conducted the tetrad analysis of the strains heterozygous for *IXR1, DUN1,* and *CLB1* genes and presented the data in Figure S4. The *clb1∆* mutation was not able to suppress the lethality of the *dun1∆ ixr1∆* double mutant. Thus, we conclude that the increased expression of *CLB1* is not the cause of the lethality. Regarding the data, the physiological significance of the control of *CLB1* or *CLB2* by Ixr1 may be different. The description of the tetrad analysis results was included in lanes 327-329, page 15, and we discussed the results in the discussion section of the revised manuscript.

**Lines 288-291: DNA damage is being indirectly assessed by RNR3 expression. It would be preferable to directly evaluate DNA damage. If not, it may be better to move this statement to the Discussion section. Since there are many repeated sections in the Results and Discussion, combining these sections into one as "Results and Discussion" might be more effective.**

According to the reviewer’s suggestion, we moved the sentences into discussion section about the speculation of the occurrence of DNA damage response from *RNR3* expressions. Moreover, we only described objective data and evoked hypothesis in the results section, and all subjective interpretations were moved to discussion section.

**Lines 297-299: In Figure 13A, the growth of dun1Δ clb5Δ appears slow, but dun1Δ clb6Δ shows little change in growth compared to dun1Δ and clb6Δ.**

We carefully evaluated the tetrad analysis results and concluded that the *dun1∆ clb6∆* double mutant grew as well as the wild-type strain. The results were described in lanes 376-378, page 17, of the revised manuscript.

**Lines 352-353: In Figure 9A, CLB1 overexpression does not appear to suppress the growth defect of puf5Δ clb5Δ. Thus, CLB1 may not compensate for CLB5. It would be important to examine whether CLB1 overexpression can suppress the growth inhibition of puf5Δ clb5Δ clb6Δ. Alternatively, the effect of CLB1 overexpression on the growth and temperature sensitivity of puf5Δ clb5Δ should be more accurately evaluated.**

According to reviewer’s point, we discreetly evaluated the results shown in revised Figure 10A and reasoned out that the multi-copy *CLB2* did not suppress the growth defect of the *puf5∆ clb5∆* double mutant. From these results, Clb1 seems not to function as a substitute for Clb5 as reviewer pointed. We described the interpretation of the results in lanes 295-302, page 14, of the revised manuscript.

**Why can Clb2 compensate for Clb5 and Clb6? A discussion of the physiological significance of this compensation would be desirable.**

According to reviewer’s suggestion, we discussed our hypothesis why overexpression of G2/M-phase cyclin Clb2 can compensate for Clb5 in lanes 435-451, page 20, of the revised manuscript.

**The figures appear somewhat disorganized, so it would be better to reconsider the integration and order of the figures to match the manuscript.**

We carefully arranged the figures in the order in which they are mentioned in the revised manuscript.

**Line 204: The statement that the puf5Δ clb3Δ clb4Δ triple mutant did not show any growth delay could be softened, as Supplemental Figure 2 seems to show a slight growth retardation. A phrase like "showed slight growth retardation" would be preferable.**

In line with the reviewer’s indication, we reinterpreted the tetrad analysis data. We presented the results in the revised Figure S2 as “the *puf5∆ clb3∆ clb4∆* triple mutant showed only a slight growth retardation” in lanes 224-227, page 11, of the revised manuscript. Since the *puf5∆* mutation caused much severe growth defect in the *clb5∆ clb6∆* double mutation background than in the *clb3∆ clb4∆* condition, we assume that the *CLB2* expression is vital for cell survival in the deficiency of S-phase cyclins.

**An explanation of what DUN1 is should be provided not only in the abstract but also in the main text, particularly in the Results section.**	We included the explanation of Dun1 in lanes 314-319, page 15, in the results section of the revised manuscript.

**The notation of genotypes should be consistent between the manuscript and the figures. For example, in the manuscript, clb1Δ clb5Δ clb6Δ is used, but in Figure 6, clb5Δ clb1Δ clb6Δ is written.**

In response to reviewer’s pointing, we thoroughly checked all of the notation of strains in the manuscript and figures and revised inconsistent notions.

**Figures 4A and B are not explained in the manuscript.**

We thoroughly checked the manuscript and explained all shown data in the revised manuscript.

**Are the cells in Figure 6 in a bar1Δ background? If so, the description in both the figure and the manuscript should be revised accordingly.**

For the experiment for the revised Figure 8, we utilized *BAR1*+ strains. Previously, it was reported that *BAR1*+ strains can also be used for cell cycle synchronization procedures by using 100-fold higher concentration of α-factor (in reference 27). We also confirmed that cell cycle synchronization was successfully performed in the *BAR1*+ *clb1∆ clb5∆ clb6∆* triple mutant and the *puf5∆ clb1∆ clb5∆ clb6∆* quadruple mutant by morphological changes and nuclei behaviors shown in Figure S3. We included the information about the strain background in lanes 236-239, page 11, of the revised manuscript.
